# Leveraging Infrastructure BIM for Life-Cycle-Based Sustainable Road Pavement Management

**DOI:** 10.3390/ma16031047

**Published:** 2023-01-24

**Authors:** Cristina Oreto, Salvatore Antonio Biancardo, Francesco Abbondati, Rosa Veropalumbo

**Affiliations:** 1 Department of Civil, Construction and Environmental Engineering, Federico II University of Naples, Via Claudio 21, 80125 Naples, Italy; 2 Department of Engineering, Parthenope University of Naples, 80133 Naples, Italy

**Keywords:** asphalt pavements, maintenance management, recycling, life cycle approach, Infrastructure Building Information Modeling

## Abstract

The latest developments in the field of road asphalt materials and pavement construction/maintenance technologies, as well as the spread of life-cycle-based sustainability assessment techniques, have posed issues in the continuous and efficient management of data and relative decision-making process for the selection of appropriate road pavement design and maintenance solutions; Infrastructure Building Information Modeling (IBIM) tools may help in facing such challenges due to their data management and analysis capabilities. The present work aims to develop a road pavement life cycle sustainability assessment framework and integrate such a framework into the IBIM of a road pavement project through visual scripting to automatically provide the informatization of an appropriate pavement information model and evaluate sustainability criteria already in the design stage through life cycle assessment and life cycle cost analysis methods. The application of the proposed BIM-based tool to a real case study allowed us (a) to draw considerations about the long-term environmental and economic sustainability of alternative road construction materials and (b) to draft a maintenance plan for a specific road section that represents the best compromise solution among the analyzed ones. The IBIM tool represents a practical and dynamic way to integrate environmental considerations into road pavement design, encouraging the use of digital tools in the road industry and ultimately supporting a pavement maintenance decision-making process oriented toward a circular economy.

## 1. Introduction

Road pavement management is a complex process that involves continuous monitoring and design of maintenance actions to keep the pavement in the desired structural and functional conditions and minimize life cycle costs [1,2].

Therefore, proper pavement maintenance planning can be regarded as a strategic approach to achieve capital spending rationalization, risk control, performance preservation, stakeholders’ satisfaction and conservation of natural resources; all these challenges should be faced efficiently at all stages of the pavement life cycle by collecting and running analytics [3].

Considering the increasing importance of delivering more sustainable and durable road pavements, the life cycle thinking approach has been regarded as an effective methodology to measure the overall sustainability of a project, both from an economic and environmental point of view, with a view on the whole life cycle from conception and design throughout the service life of the infrastructure up to dismission, demolition, disposal or recycling [4,5].

Some life cycle techniques are well established in the construction industry and especially in the field of maintenance planning and management, i.e., life cycle cost analysis (LCCA), while others, such as life cycle assessment (LCA), are still on the way to be fully integrated into common infrastructure sustainability rating systems applied to road pavement management.

In detail, with the aim of lowering the consumption of nonrenewable raw materials and fossil fuels in the construction and maintenance of asphalt road pavements, researchers are trying to quantify, through internationally consolidated and standardized LCA methodology, the potential environmental impacts and the relative benefits of alternative bituminous materials containing secondary raw materials in substitution of natural ones [6,7], bitumen modification to extend the service life of pavements [8] and low-temperature production technologies [9,10]. In addition, LCA can be applied for comparison/decision-making purposes to rate the environmental sustainability of different construction and maintenance solutions with alternative asphalt materials versus traditional hot-produced ones [11].

A common issue when applying LCA to support decision making in the field of road pavement management is that it often requires large time expenditure and data; therefore, LCAs are often performed at the end of the design phase, when most of the design configuration (i.e., geometry, materials and service life) has been already defined, leaving no other time to incorporate the environmental sustainability rating into decision making [12].

As new digital technologies are emerging in the construction sector, new tools are available to researchers to ease information management and consequent decision making by running analyses in an automated context before setting up the pavement design configuration.

As a matter of fact, the digitalization of road infrastructure projects can be supported by workflows that improve not only the quality of the delivered project but also the efficiency of their development, improving communication and data flow between project participants. More and more digital tools and technologies are supporting construction and maintenance processes of road infrastructures, such as, in the case of linear infrastructures, infrastructure building information modeling (IBIM); IBIM has been already regarded as a tool that can help in the early detection of omissions and errors, improved productivity, structure simulation and analysis and improvement of communication between the actors of the process through more informed participation and data sharing. However, the adoption of IBIM in the infrastructure field and the issues related to linear asset management still significantly hinder further developments and adaptations to fully make the most of the listed benefits [13].

Therefore, IBIM tools have recently begun to spread across infrastructure engineers to efficiently archive, store, manage and analyze large amounts of data generated by different actors and analytic tools involved in the project, ultimately aiming to support decision making in road asphalt pavement design and management [14,15]. For example, Delgado et al. [16] checked the potentialities of feeding road pavement monitoring data into an IBIM, obtaining the practical advantage to evaluate several design/management alternatives already in the pavement design phase; each change in the design/maintenance of the pavement, i.e., reinforcements, maintenance actions, alterations to the original structure or changes in survey methods, generated automatic changes in the IBIM, which were easily readable for the project participants.

Looking more specifically at the leverage of IBIM tools to integrate pavement structural and performance data, Tang et al. [17] developed a digital model of a road pavement and coded a customized script aimed at performing structural controls and checking the accumulated rutting damage at the end of the predicted service life based on empirical models fed by asphalt-mixture-specific data regarding its physical and mechanical properties. Other scientists have focused on the integration of BIM and quality control checks [18] and proposed BIM as an information integration platform to facilitate construction project management applied to infrastructures, highways and bridge projects [19].

Gradually, the need to control sustainability indicators and generate continuously relevant information related to life cycle environmental sustainability throughout the infrastructure project life cycle has found the potential application of IBIM tools, moving forward from just geometrical detail, 3D visualization [20,21] and interference detection [22] to the integration of advanced information and tailored analytics in the IBIM process to actively support design and management tasks [17].

Therefore, the full development of IBIM potential delivers a digital and smart representation of data-enriched objects created through the collaboration between the involved parties to provide feedback at the earliest possible time, improving decision-making processes, and prompt project efficiency at all stages of the life cycle [23].

Little effort has been dedicated to the full integration of IBIM potentialities with life-cycle-based methodologies to assess the environmental and cost sustainability of a road project in light of more efficient decision making.

Looking at what has been achieved in other engineering fields, Antón and Díaz [24] introduced two methodologies for the integration of BIM and LCA for geotechnical works. The first approach relies on the extraction of information from the BIM model to assess LCA impact category indicators; the main advantage is the accuracy of the results, while a drawback is the increasing complexity of the BIM model. The second approach is mostly based on manual input of the environmental performance indicators in the digital model, which does not require any programming skills or efforts but totally lacks dynamism of the information and analytic capabilities.

Looking at how researchers leveraged the information exchange to implement basic environmental sustainability assessments in the IBIM environment, Liu et al. [25] proposed a theoretical framework for introducing a sustainability infrastructure rating system into an IBIM environment that helps to set the consistency of each element’s attributes (both functional and physical) in relation to a set of sustainability thresholds imposed by the user. Kaewunruen et al. [26] enriched the information model of a bridge project with the embodied carbon footprint of the life cycle of different elements based on their constituent material identifiers, obtaining a metric of the environmental impacts of each model component that was later manually transferred from the BIM environment to the LCA tool. Lastly, Slobodchikov et al. [27] developed an Autodesk Civil3D application that focuses on the calculation of a specific impact category indicator, namely, the global warming potential (GWP), of a road asphalt pavement structure based on the material volumes of each layer and the construction operation scheduling gathered from the information model, also returning a visual representation in a color scale of the GWP of each element of the road pavement.

Although the BIM/IBIM research has shown efficient possibilities to customize projects with detailed and automated analysis tools to integrate sustainability rating and criteria already in the design stage (e.g., selection of materials, prediction of structural performance and required maintenance actions, visualization of environmental impact indicators and cost minimization), a comprehensive effort has not been dedicated yet to (a) the depiction of a life-cycle-based sustainability framework specifically designed for IBIM of road pavement projects and (b) the integration of both the aspects of sustainability assessment/rating and planning of future maintenance interventions according to rational decision-making criteria within the IBIM of a road pavement.

**Figure 1:**
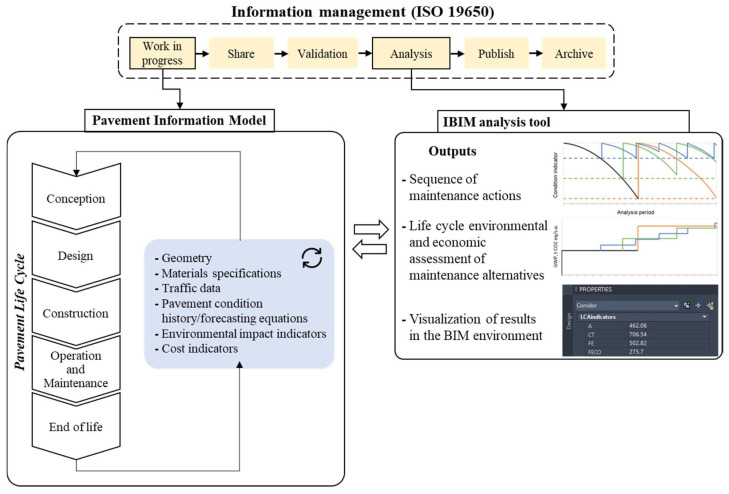
Structure of the designed IBIM tool in the ISO 19650 information management framework.

For these reasons, the present research aims to fill the following gaps:

- Definition of a decision support system oriented to reactive and predictive maintenance, which considers the variables related to economic and financial aspects up to the environmental and technical–operational ones related to the decay of specific status indicators; the framework must be compliant with ISO 19650 information management protocols (see Figure 1) and is aimed at automating the drafting of a multiyear maintenance plan for a road pavement section.
- The introduction of laboratory results related to road construction materials (i.e., composition of the mixtures and features of primary and secondary raw materials, as well as the physical, mechanical and performance-related attributes and predictive equations useful for designing and predicting the service life of asphalt pavement configurations) into a BIM workflow.
- Digitization of the road pavement management process through the definition of a pavement information model aimed at IBIM-based sustainable maintenance management. Integration is intended not only for the automation of data management but also for the definition of specific information exchange management protocols related to the life cycle of a road pavement.

The novelty of the study mainly consists of filling the knowledge gap on the use of IBIM as a decision-making tool for the selection of pavement design configurations to be applied during the construction/maintenance process according to multiple sustainability criteria; to reach this goal, a theoretical framework has been developed and subsequently coded as an IBIM plugin.

The main practical–applicative feedback and relevant deliverable of the present work is the IBIM plugin in support of the designers and the road management bodies themselves, which interacts with the informative content (geometric, functional, structural, cost and environmental-related parameters) of an IBIM pavement digital model, supported by continuous and updated flows of data related to the monitoring of the pavement in situ, to provide instant, automated and continuous prediction of the performance, costs and environmental impacts of the life cycle.

## 2. Methodology

The present section focuses on the main methodologies applied to design the methodological framework on which the actual IBIM-based analytical tool relies, also in light of the definition of a proper pavement information model.

### 2.1. Condition Indicators and Alternative Maintenance Strategies

First, the condition indicators, their thresholds and consequent maintenance actions were set up.

During the service life of a pavement, its conditions should be accurately evaluated to identify the severity of pavement damages and types of pavement distress. Therefore, monitoring systems are considered a significant step in the maintenance processes.

A typical document based on which visual assessments of pavement distresses are made is the Distress Identification Manual for the long-term pavement performance program [28].

The type of pavement distresses and their degree of severity and extension concur to a single global indicator of pavement condition, i.e., the PCI, a widely used index derived from individual distress deduct values developed in the late 1970s by the U.S. Army Corp of Engineers [29]. It provides a measure of the current condition of the pavement based on the distresses observed on the surface and is intended to be an indicator of a pavement’s structural integrity and surface operational condition (i.e., localized roughness and safety). The type and severity of pavement distress are assessed by visually inspecting pavement sample units, and each distress indicator contributes to the overall condition of the sample unit, whose aggregation depends on the homogeneity of pavement condition. The rating scale of the PCI indicator ranges from the minimum value, 0, to the maximum value, 100, with 100 representing a pavement in perfect functional and structural condition.

The reactive maintenance approach involves the continuous definition of the maintenance activities as a result of the distress surveys carried out through time; corrective measures are only initiated after clear pavement distress or other deficiencies in road condition have been identified [30]. Instead, preventive maintenance aims to apply a series of low-cost preventive treatments whose main objective is to increase the service life of pavements before reconstruction is needed [31]; the proper timing for the application of the corrective measures can be achieved by predicting the residual service life of the pavement before failure occurs (i.e., bottom-up fatigue cracking).

Disregarding any rational approach (i.e., reactive or preventive/predictive) to road pavement maintenance will result in what is often addressed as “urgent maintenance”, in which deep repairs are carried out after an event occurs that cannot be foreseen but requires immediate attention due to user safety concerns [32]. The aforementioned maintenance approach often results in the poorest pavement condition and high life cycle costs and environmental impacts, as less frequent and deeper maintenance actions (i.e., rehabilitation and reconstruction) are applied without further extending the service life of the pavement [33].

**Figure 2:**
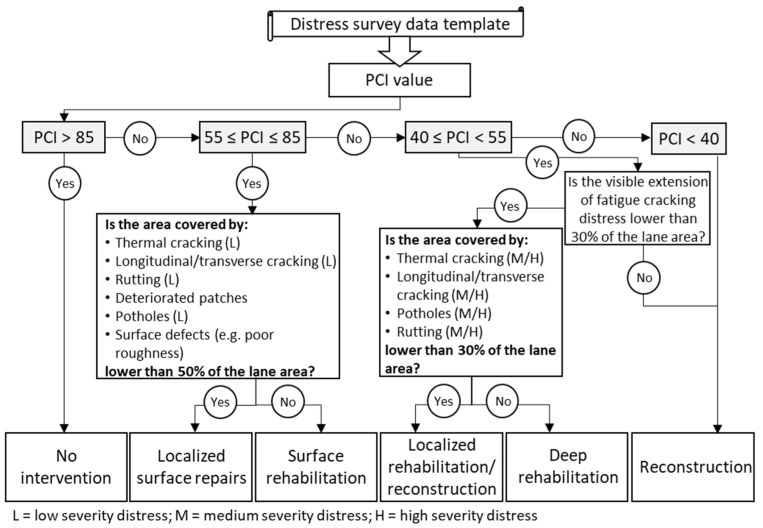
Structure of the reactive maintenance algorithm, PCI thresholds and relative maintenance actions.

Aiming to set up an automated reactive maintenance algorithm, a rigid set of rules and actions was originally developed in the present work based on the type, severity and density of each distress identified according to the Distress Identification Manual for the long-term pavement performance program [28]. In detail, Figure 2 shows the logical flow implemented in the maintenance algorithm, starting with the evaluation of the distress categories and ending once the proper maintenance strategy is identified. As reported in Figure 2, the choice of the proper maintenance strategy takes into account the traditional PCI thresholds [34]; additionally, the type, extension and severity of certain distress categories are taken into account to assess whether the specific maintenance intervention should be applied to the whole pavement surface or limited to a portion of the surface to resolve localized failures (e.g., patching and sealing) and restore a high PCI value over the whole pavement surface.

In particular, three main maintenance interventions were triggered by the PCI values (see Figure 2):

- Surface rehabilitation: it implies the milling and reconstruction of the wearing course and the assessment of the condition of the binder layer (i.e., extraction of core samples from the binder layer to test the stiffness modulus, in situ testing to measure the bearing capacity of the deeper layers, etc.);
- Deep rehabilitation: it consists of the milling and reconstruction of the wearing course and binder layer (and relative tests applied to measure the structural capacity of the base layer, i.e., coring of the base layer and stiffness measurement);
- Reconstruction: it involves the full reconstruction of the asphalt layers (wearing, binder and base layer) and subsequent control of the subbase bearing capacity.
- PCI values above 85 imply no maintenance interventions due to the good quality of the pavement surface.

Once the type of maintenance action is defined, a proper pavement library must be set up to choose from alternative design configurations.

Road flexible pavement design is a fundamental step to ensure the compatibility of the materials, pavement geometry and subgrade conditions with the loads expected during the useful life of the pavement. Ultimately, pavement design aims to define the optimum thickness of the pavement layers, given the structural model as an elastic, homogeneous and isotropic multilayer and several input data, such as the mechanical performance of the materials used for each layer (i.e., constitutive law and Poisson ratio) and the design service life, to prevent excessive extension of fatigue cracking damage and rut depth [35]. A standardized way to characterize the stiffness of asphalt mixtures in the laboratory is the indirect tensile test as reported in EN 12697-26-Annex C. This method measures the resilient stiffness of bituminous mixtures using an indirect tensile test by applying load with a haversine waveform.

The target of pavement design is to ensure that specific distress indicators, such as fatigue cracking and rutting, are contained below certain predetermined thresholds.

Fatigue cracking is a typical distress mechanism that occurs in flexible pavements; it starts at the bottom of the asphalt base layer and propagates to the surface as one or more interconnected cracks. An excessive extension of the area of the pavement affected by fatigue cracking may lead not only to reduced ease and safety in driving but also to the percolation of hazardous substances from the bonded layers to the soil and underground waters, especially when recycled materials are added to the asphalt mixtures [36].

The cumulative fatigue damage (*FD*), which must be kept below 1 to avoid excessive deterioration of the surface quality (*FD* equal to 1 means that 10% of the lane area is covered in cracks), is determined as the sum of the seasonal share of relative damage produced by the ESALs passings during the *i*-th season, according to the Miner law, which is reported in Equation (1).

(1) $$FD=\sum_{i=1}^{S}\frac{n_{i}}{N_{i}}$$

where *n_i_* is the number of ESAL passages in the *i*-th season, which is computed through the AASHTO Design method [37] supposing that the ESAL passings are evenly distributed between the four seasons, and *N_i_* is the number of ESAL passages that leads to an extension of fatigue cracking damage up to 10% of the lane area of the road pavement, determined according to the Asphalt Institute fatigue prediction law [36] through the average ITSM and the tensile strain at the bottom of the base layer resulting from the linear elastic multilayer model.

Besides fatigue cracking, rutting is another typical distress phenomenon that often occurs in asphalt pavements and consists of the accumulation of permanent deformations in the asphalt layers, resulting in a depression of the surface along the wheel path. Hazardous conditions on roads can occur because of rutting. The depressions are known to hold water, which can cause the tires to lose contact with the road surface, decreasing road safety, and possibly cause infiltration to groundwater, especially if combined with cracks on the pavement surface [38].

The rut depth (R) at the end of the service life, which should never be deeper than 2 cm for Italian suburban roads, is predicted through the Vaerstaten law, as shown in Equation (2).

(2) $$RD=\sum_{j}\sum_{i}\varepsilon_{ij}\cdot h_{j}$$

where *ε_ij_*∙ is the permanent deformation in the *i*-th season that is accumulated in the *j*-th asphalt layer of the pavement after the mentioned ESAL passings, determined according to the Kaloush and Witczak model [38] based on the vertical compressive strain obtained from the linear elastic multilayer model, and *h_j_* is the thickness of the *j*-th asphalt layer.

The set of maintenance alternatives included, besides the conventional HMA, modified asphalt mixtures (polymer-modified asphalts (PMAs), conventional asphalt mixtures with polymer-modified bitumen (PMB) and HMAs with secondary raw materials in substitution of natural aggregates, as well as sustainable solutions such as in-place recycled cold mix asphalts (CMAs) for either the binder or the base layers.

The prediction of the pavement condition over time was carried out through decay curves, such as those based on the mentioned empirical fatigue and rutting damage accumulation laws, but also, where historical PCI series of data were available, through the interpolation of these data. Each of these parameters was associated with one or more threshold values corresponding to the need for superficial, deep rehabilitation or full reconstruction. Then, for each maintenance strategy, the number of years after which a maintenance intervention will be necessary was identified as the minimum number of years required to reach the respective limit condition for each condition indicator using Equations (3)–(5) for the surface rehabilitation, deep rehabilitation or reconstruction intervention.

(3) $$N_{SR}=N_{SR,PCI}$$

(4) $$N_{DR}=\min\left(N_{DR,PCI};N_{DR,U}\right)$$

(5) $$N_{RE}=\min\left(N_{RE,PCI};N_{RE,FD}\right)$$

where

$N_{SR}$ is the number of years before the next surface rehabilitation intervention, which is equal to the number of years before reaching the predicted PCI value of 85 ($N_{SR,PCI}$);

$N_{DR}$ is the number of years before the next deep rehabilitation intervention, which is selected as the minimum between the number of years before reaching either a PCI value of 54 ($N_{DR,PCI}$) or an accumulated rut depth equal to 2 cm ($N_{DR,U}$);

$N_{RE}$ is the number of years before the next reconstruction intervention, which is selected as the minimum between the number of years before reaching either a PCI value of 39 ($N_{RE,PCI}$) or an accumulated fatigue damage value equal to 1 ($N_{RE,FD}$).

### 2.2. Environmental and Economic Assessment of Alternatives

The LCA procedure is internationally standardized by the EN ISO 14040 and 14044 standards [39,40]. The LCA (as defined by the ISO 14040 standard) considers the environmental impacts of the case examined on human health, the quality of the ecosystem and the depletion of resources. The objectives of this methodology are to define a complete picture of the interactions with the environment of a product or service, helping to understand the environmental consequences directly or indirectly caused and therefore providing those who have the decision-making power (who have the task to define the regulations) the information necessary to define the behaviors and environmental effects of an activity, and identify opportunities for improvement in order to reach the best solutions to intervene for the reduction in environmental impact. In this study, the impact assessment of the designed pavement solutions was performed through SimaPro 9^®^ software. Among the impact assessment models available in the literature, the Egalitarian ReCiPe [41] impact assessment method was selected both for its diffusion in the construction sector and for the number of environmental problems quantified by its impact category indicators, namely, 18 midpoint indicators and 3 endpoint indicators. Midpoint- and endpoint-level indicators refer to different phases in the cause–effect chain that start from the inventory flows and are converted into midpoint effects on specific environmental topics, which, at the end of the cause–effect chain, produce effects on broader endpoint impact categories, such as human health, ecosystems and resource availability. The following midpoint impact categories were considered: climate change (GWP, kg CO_2_ eq); stratospheric ozone depletion (ODP, kg CFC11 eq); ionizing radiation (IR, kBq Co-60 eq); damage of ozone formation on terrestrial ecosystems (OFT, kg NO_x_ eq) and human health (OFH, kg NO_x_ eq); fine particulate matter formation (PM, kg PM2.5 eq); terrestrial acidification (A, kg SO_2_ eq); freshwater eutrophication (FE, kg P eq); marine eutrophication (ME, kg N eq); terrestrial, freshwater and marine ecotoxicity (T-ECO, F-ECO and M-ECO, kg 1,4-DCB eq); human carcinogenic (CT, kg 1,4-DCB eq) and noncarcinogenic toxicity (NCT, kg 1,4-DCB eq); land use (LU, m^2^ a crop eq); mineral resource scarcity (MR, kg Cu eq); fossil resource scarcity (FR, kg oil eq); and water consumption (W, m^3^).

Table 1 summarizes all the adopted equations to make automatic evaluations of the life cycle sustainability indicators of the pavement structure under analysis.

The cost of road construction consists of design expenses, material extraction, construction equipment, maintenance and rehabilitation strategies and operations over the entire service life.

The American Association of State Highway Officials (AASHO) introduced the concept of life cycle cost–benefit analysis in its “Red Book” in 1960. The LCCA was introduced to support highway investment decision making and economic evaluation of highway upgrades during the planning stage. The use of the LCC concept is supported in the different AASHTO Pavement Design Guide editions [37,42], which also include detailed discussions regarding costs that should be considered in LCCA and discount methods [43,44].

Table 2 summarizes the adopted equations to make automatic evaluations of the life cycle costs of the pavement structure under analysis.

### 2.3. Decision Making

Multiple attribute decision making (MADM) involves “making preference decisions (such as evaluation, prioritization and selection) over the available alternatives that are characterized by multiple and usually conflicting attributes” [45,46].

An MADM problem is depicted as a decisional problem that involves a predetermined set of alternatives, each one described with a specific set of attributes/indicators. An MADM problem generally requires three methodological steps to be solved:

- Determining the weights of the attributes;
- Normalizing the attribute values for each alternative;
- Aggregating the normalized attribute values into an overall index to produce the ranking of the alternatives [47].

In MADM problems, weight commonly defines the relative importance of an attribute within the set of attributes that are available to the decision-maker. Equally weighting the attributes is the simplest way to perform MADM as it requires minimal input from decision-makers [48,49].

In the present study, a total of 22 indicators, the sum of 18 environmental impact indicators, 1 life cycle cost indicator and 3 performance/damage indicators, were used as the criteria of the analysis, and the alternatives of the analysis were, in the first case, 12 alternative reconstruction solutions and 4 rehabilitation alternatives.

The decision matrix *A* was obtained considering the set of maintenance alternatives $M_{1}=\left\{1,\ldots j,\ldots m\right\}$ with *m* = 16 and the set of criteria $I=\left\{1,\ldots i,\ldots l\right\}$ with *l* equal to 22, as shown in Equation (6).

With the entry *a_ij_* of A, we refer to the value of the *i*-th criteria related to the *j*-th alternative maintenance solution.

(6) $$\underset{22\times 12}{A}=\left(\begin{array}{ccc} a_{1,1} & \ldots & a_{1,12}\\ \vdots & a_{ij} & \vdots \\ a_{22,1} & \cdots & a_{22,12} \end{array}\right)$$

Since all the criteria reported into the decision matrix has different units of measurements, normalization was carried out to obtain the normalized decision matrix *N* (see Equation (9)). For the criteria where a high value represents the best performance (PCI), the normalization occurred by using Equation (7), while in the case of R, FD and LCC indicators and the 18 environmental impact indicators, where the lowest values represent the best performance, the Equation (8) was adopted.

(7) $$n_{1,ij}=\frac{a_{ij}}{\max\;a_{i}}$$

(8) $$n_{1,ij}=\frac{\min\;a_{i}}{a_{ij}}$$

where $n_{ij}$ is the normalized *i*-th criteria in the range [1;22] for the *j*-th maintenance solution and $\max a_{i}$ and $\min a_{i}$ are the maximum and minimum values of the *i*-th criteria, respectively, among all maintenance solutions.

(9) $$\underset{22\times 16}{N}=\left(\begin{array}{ccc} n_{1,1} & \ldots & n_{1,16}\\ \vdots & n_{ij} & \vdots \\ n_{22,1} & \cdots & n_{22,16} \end{array}\right)$$

Finally, each *i*-th maintenance alternative is assigned a synthetic score based on the utility method [50], equal to the sum of the products between the normalized value of the *j*-th indicator and the weight assigned to the *j*-th indicator (see Equation (10)).

(10) $$U_{i}=\sum_{j}w_{j}\cdot n_{i,j}$$

where $U_{i}$ is the final score of the *i*-th alternative according to the utility method, $w_{j}$ is the weight (importance) assigned to the *j*-th indicator and $n_{i,j}$ is the normalized value of the *j*-th indicator of the *i*-th maintenance alternative.

## 3. Results and Discussion

Before the actual coding of the analysis tool, a careful pavement information model was defined considering the expected life cycle management results. Then, the main explanatory variables of the maintenance alternatives required careful and broad data collection. All the data related to the application of the present methodology to a case study are collected in the Supplementary Materials as follows:

- Tables S1 and S2 collect the mix composition data of the asphalt mixtures under analysis, respectively, for the binder and base layers;
- Table S3 collects the asphalt stiffness moduli at the service temperature used for pavement design; more on the experimental mix design procedure and performance characterization of the asphalt mixtures under analysis can be found in Oreto et al. [51] and Russo et al. [52];
- Table S4 collects all the available data regarding the road category, the AADT and the expected number of ESALs in 20 y for the case study under analysis, on which pavement design was based;
- Table S5 summarizes the results in terms of the type of maintenance intervention, expected frequency of intervention and materials involved;
- Table S6 collects all the data sources and relative survey year of LCA data;
- Table S7 summarizes all the cost items of the alternative maintenance strategies;
- Table S8 reports the final decision matrix of the case study.

The IBIM framework encompassed three main steps, as follows:

- Set up data templates (i.e., Excel spreadsheets) to import the needed information in the programming interface and speed up the informatization of the pavement information model;
- Informatize the IBIM of a road pavement through property sets definition;
- Run calculations and update the property sets with the outcome of the maintenance algorithm and decision-making framework.

Taking into account the engineered maintenance algorithm, a visual programming tool (namely, Dynamo, an open-source add-in for Autodesk applications [53]) was leveraged to implement further sustainability and maintenance analyses in an IBIM project.

“Visual Programming Language” is a concept that provides designers with the necessary means to construct unique relationships between digital objects using a simple graphical user interface. Rather than coding from scratch, the user can assemble existing custom relationships by connecting prepackaged nodes together to make a custom algorithm. The main consequence is that designers, who do not usually have developed coding skills, can implement computational concepts and enrich their projects with targeted calculations.

Dynamo allows designers to automate processes, perform data manipulation, implement relational structures and analytic capabilities and control Vasari Families and Parameters, which would not be usually possible without a conventional modeling interface. Last but not least, Dynamo offers the designer the opportunity of doing so within the context of an IBIM environment.

**Figure 3:**
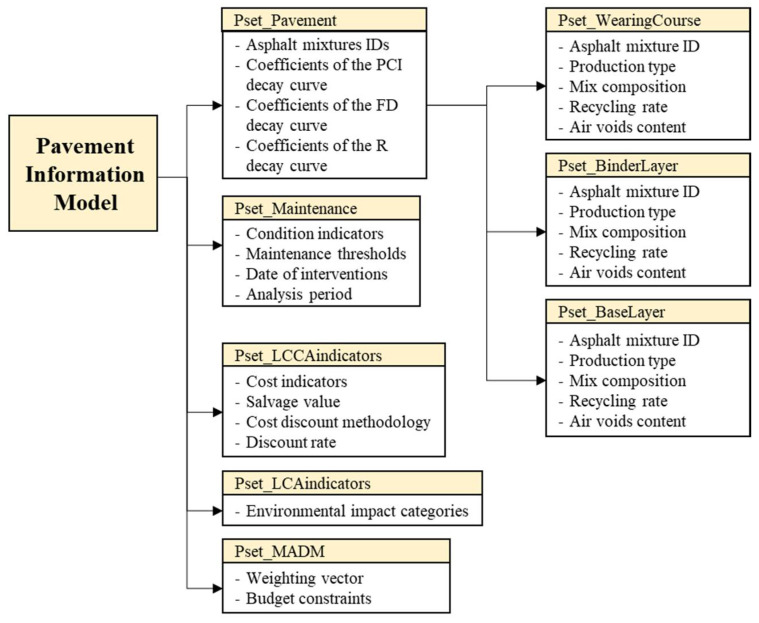
Structure of the pavement information model oriented to sustainable maintenance.

At this point, the IBIM of the road pavement (Civil 3D [53]) was fully informatized using a combination of visual scripting and Python scripting to retrieve the necessary information during each step of the algorithm. Additional alphanumeric information was assigned to the digital 3D solids of the road pavement, in addition to the geometric features of the parametric pavement section extruded over the road alignment; this information was collected into property sets, namely, custom sets of parameters associated with a certain object that can be easily accessed by the user in the Extended Data Table. Figure 3 shows a schematic representation of the pavement information model.

Each property, belonging to a specific property set, can be regarded as:

- Input property: its value is assigned directly from the data template imported by the user and does not require additional calculations;
- Output property: its value is the result of the calculations of the analytic tools supporting the pavement IBIM.

The following property sets were implemented into the IBIM environment (see Figure 3):

- Pset_Pavement: the property set includes the current features of the asphalt pavement, such as the asphalt mixture identifiers of each layer and the coefficients of the PCI, FD and R decay curves;
- Pset_WearingCourse, Pset_BinderLayer, Pset_BaseLayer: the three input property sets, each one attached to the respective asphalt layer of the pavement structure, include all the necessary information that should be uploaded in the BIM environment before performing the LCA in absence of a specific EPD;
- Pset_MADM: the property set includes the input parameters that set up the boundary conditions for MADM (the weighting coefficients of each indicator and the maximum budget constraint in the analysis period);
- Pset_Maintenance: the property set includes both input (analysis period) and output parameters (type of maintenance strategy, number of years before next maintenance intervention, relative trigger condition and value of each condition indicator before next maintenance intervention) referred to the current pavement configuration;
- Pset_LCAindicators: the output property set includes the environmental impact categories that will be filled once the analysis tool is run on the current pavement configuration. In particular, hierarchical recipe midpoint impact assessment methodology [41] was chosen to address 18 different environmental problems through as many impact category indicators;
- Pset_LCCAindicators: the property set includes both input (discount rate) and output parameters (LCCA indicator and salvage value of the pavement at the end of the analysis period) used to characterize the life cycle cost dimension of the current pavement configuration.

Since the creation of property sets is time-consuming and could easily lead to errors in structuring the data, the command block embedded into the Dynamo programming interface was leveraged to automate the creation and upload of the parameters into the BIM environment.

From the point of view of the integration of IBIM and life cycle analyses, Dynamo, a Civil3D extension that creates a dynamic link between the BIM environment and an open-source visual programming environment, was leveraged to equip the pavement BIM with additional analytic tools that run calculations and update the values of the object properties with the outputs of the calculations.

**Figure 4:**
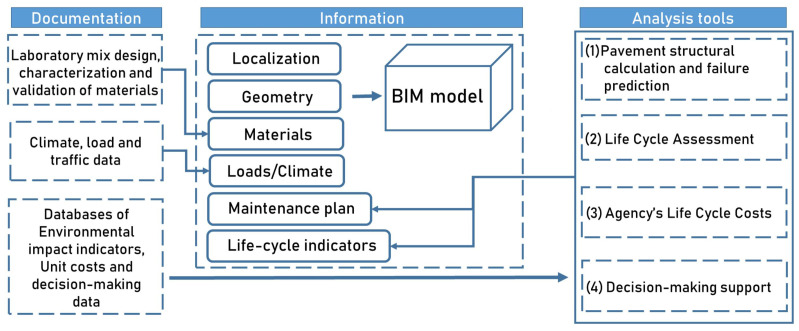
Schematic overview of the information collection, exchange and generation through analytic tools.

A schematic overview of the designed analytic tools is reported in Figure 4.

**Figure 5:**
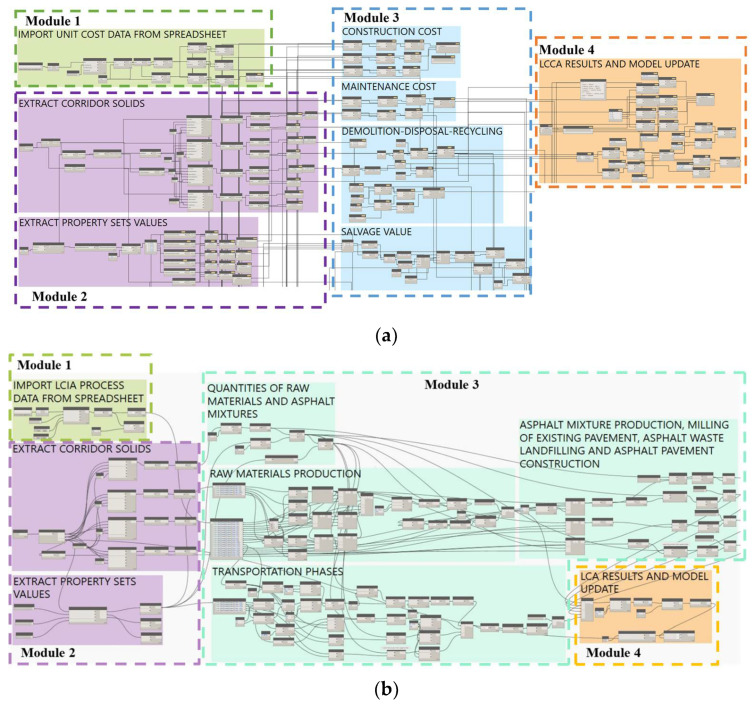

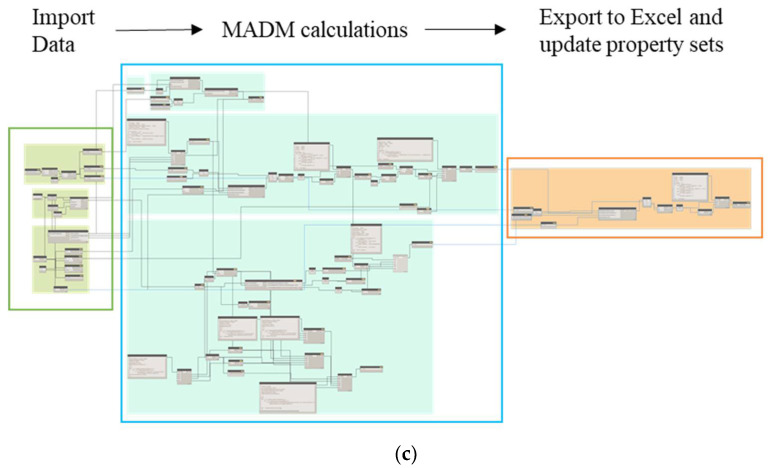
Appearance of the visual programming coding in the Dynamo environment to (**a**) run LCCA calculations; (**b**) run LCA calculations; and (**c**) run MADM calculations.

The analytic tools (which are Dynamo files with .dyn extension) must be executed in series to produce the desired decision-making result and are as follows:

- The PMS tool gathers the information (time series of PCI surveys, predictive rutting and fatigue accumulations laws, pavement solutions library, intervention thresholds, etc.) and calculates the type and timing of the maintenance interventions according to different maintenance approaches;
- The LCA/LCCA (see respectively Figure 5a,b) tool gathers the outputs of the PMS tool, as well as the libraries of unit costs and impact category indicators for each stage of the expected life cycle of the pavement, to calculate a set of life cycle indicators for each alternative pavement solution;
- The MADM (see Figure 5c) tool applies the budget constraints and performs the final decision making based on the decision matrix set up by the execution of the previous analysis tools. The MADM tool also updates the final values of the output properties, including the number of years before the next intervention, the condition indicator that triggers the need for maintenance, the cost and the environmental impact indicators of the life cycle of the optimal solution.

Each module of the algorithm also includes the production of reports, exported as spreadsheets, such as the time evolution of the condition indicators for each unit sample and the timing, type, cost and environmental impacts of each maintenance strategy for each unit.

### Application to a Case Study

The elaborated procedure was applied, and its effectiveness was tested in a real case study, in which IBIM was used as a decision support system to choose the optimum maintenance intervention and draft future maintenance plans based on the available life cycle and performance data at the time of the analysis. The reference analysis period of the case study is equal to 50 y, and all the collected data are reported in the Supplementary Materials.

The case study under analysis refers to an existing section of a rural road in the Campania region (Italy) for which visual surveys of the type, extension and severity of distresses (and consequent PCI determination) have been carried out every 2 years for the past 6 years. The paper-based documentation regarding the geometry of the 1 km length existing road pavement section was collected and digitized to obtain the 3D digital model that was later enriched with the results of the decision-making framework.

Additional data available to the managing road administration, such as the AADT and the predicted number of ESALs in 20 y, have been collected and reported in Table S4 in the Supplementary Materials.

The current section briefly shows the results as they are obtained by feeding the information (collected into predetermined data templates) into the IBIM environment and running the BIM analysis tools.

The final decision matrix obtained by running the algorithm in the IBIM environment is reported in Table S8.

Looking in detail at LCA results (data sources used to fill the life cycle inventory reported in Table S6), concerning the GWP, the combination of a polymer-modified asphalt mixture for the base layer with a cold in-place recycled base layer lowers the GWP by 35% compared to that of the traditional reconstruction solution with HMA; the reason is the combination of using a high rate of recycled materials in the base layer, which lowers the overall amount of greenhouse gases emissions (e.g., CO_2_, CH_4_, and NMVOC) from both industrial facilities and transportation of virgin raw materials to the asphalt plant, and the satisfactory performance in terms of FD and R accumulation, as well as the service life of the designed pavement. On the other hand, the traditional rehabilitation strategy with HMA both in the binder and wearing course does not provide satisfactory results in terms of GWP (+33% compared to the traditional reconstruction strategy with HMA); the underlying reason is the high frequency of maintenance interventions to keep the R condition index under the predetermined threshold of 20 mm depth, which entails a high consumption of virgin resources, fossil fuels and energy.

Despite the considerable variability in terms of functional units, geographical context and impact assessment methods, the present LCA results can be supported by those reported in the existing literature. For example, similarly to what Yao et al. [54] found, the avoided GWP of disposing waste plastic at landfills was not as significant as the avoided impact from virgin materials production and service life extension; additionally, the most significant GWP reduction was achieved through the substitution of virgin aggregates with RAP, with −37% GWP using 40% RAP in [54] and −25% GWP using 70% RAP in the base layer in the present work.

The calculation of the LCCA indicator for the construction and maintenance operations of the asphalt layers of a flexible road pavement was implemented as a visual script within Dynamo software (Dynamo for Civil 3D 2022 software), which is able to dynamically interact and exchange information regarding, among others, the chosen design solution, in terms of layer thicknesses and the main features of the asphalt materials and the volumes of materials needed for the specific road works.

The data collection for the calculation of the unit prices of the asphalt materials was carried out by analyzing the market prices of several local companies producing limestone and basaltic aggregates and asphalt mixtures, as well as companies carrying out road works that have already experimented with the cold in-place recycling technology procedures, other than the consultation of up-to-date price lists of regional public works (see LCCA results in Table S7 in the Supplementary Materials).

The results of the life cycle costs borne by the managing agency are reported in Table S7, with reference to cost items related to the manufacturing, construction and maintenance process of the bituminous solutions; in detail, both the LCC indicator without considering the salvage value of the pavement and the net LCC indicator are reported in the output datasheet generated by the BIM analysis tool.

The LCC results show that the minimum cost strategy entails using the PMA and HMAJ, respectively, for the binder and base layer, maximizing the service life and lowering the maintenance cost by 47% compared to those of the baseline reactive strategy with traditional HMA; similar results have been found by Hasan et al. [55], who calculated a 43% reduction in the maintenance cost when using up to 25% recycled aggregates in the base and binder layer of a pavement section compared to a virgin HMA. Additionally, as reported in Table S7, the longer the service life of the maintenance treatment, the lower the impact on the life cycle costs of the materials production stage [56].

Looking at the decision-making phase, in the present research, four different alternatives have been designed, each one better than the other with respect to a specific dimension: for example, the solutions with CRMA as a base layer are better than the others in terms of environmental dimension and costs of construction but can be outperformed by stiffer solutions such as HMAJ when it comes to the maintenance phase. To take into account all the dimensions for the decision-maker to choose “the best” solution among the range of feasible solutions to adopt across a 50-y analysis period, MADM has been adopted as a comparative evaluation method.

Before defining the weight vector for MADM, the 22 criteria were collected into three subgroups:

- The performance indicators at the end of the analysis period, namely, FD, R and PCI (where available), make up the overall pavement condition (PC);
- The LCC indicator alone represents the costs incurred by the road managing agency in the analysis period (LCCA);
- The 18 indicators obtained by LCA analysis were collected to constitute the environmental and human health performance of the asphalt mixtures (EHP).

The final weight vector was defined by distributing the weight evenly among each group.

The best alternative that makes the most of the three groups of evaluation criteria is the reconstruction strategy with HMAC as the binder layer and CMRA as the base layer. In contrast, the alternatives with the traditional HMA as the base layer often qualify as the least performing, cost-effective and sustainable alternatives in the analysis period.

A large number of examples are available in the scientific literature validating the use of MADM in pavement management problems, i.e., investment planning [57], prioritization of maintenance sections [58] and choice of recycling technique [59]. Comparing the present work to the literature results, a common trait is that the different spheres of sustainability (i.e., technical, economic, environmental and, in some cases, social) have been expressed through synthetic indicators to ease the weighting of criteria and interpretation of results [56,59,60]. As for studies [61,62], only limited alternatives are included in the set of asphalt pavement maintenance activities and no rational criteria are reported for service life calculation and prediction of future pavement condition, which were the fundamental criteria of the present decision-making process.

**Figure 6:**
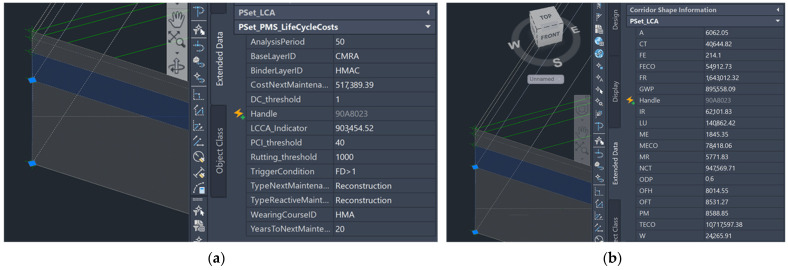
Visualization of the information generated by the IBIM analytic tools into the digital model of the pavement: (**a**) LCA indicators; (**b**) LCC indicator.

One of the expected results in terms of IBIM information enrichment is the visualization of the results of the algorithm for each element of the pavement structure in an automated and dynamic way. As shown in Figure 6a,b, the outputs of the analyses are assigned to some automatically created property sets that are either associated with a specific layer or refer to the whole road corridor, such as LCA and LCC indicators and the results of the maintenance algorithm. After running the scripts, the mentioned property sets were filled with the results: Figure 6a,b show the final information assigned to each property set, respectively, the LCA indicators and LCC indicator, together with the maintenance and MADM results.

## 4. Conclusions

With the concepts of sustainability and life cycle thinking becoming more important in the field of asphalt pavements and road pavement maintenance, the present work provided an effective framework to approach the use of IBIM platforms to provide additional analysis tools oriented towards sustainability in the field of road asphalt pavements, helping to assess the potential environmental impact and costs borne by the managing bodies to perform construction/maintenance treatments, expressed through multiple environmental and cost indicators calculated according to LCA and LCCA methodologies. The proposed tool expands the current knowledge on IBIM-LCA-LCCA integration oriented towards the promotion of sustainable pavement maintenance practices through tailored decision-making processes.

Once the IBIM-based analysis tool was run, the environmental and cost assessment returned by the IBIM analysis tool allowed the in-depth analysis and comparison of several alternative road construction materials: the main source of variability of the environmental impact category indicators was the adoption of the cold in-place recycling technology for the reconstruction of the base layer, which lowered all the impact category indicators: on average, −22% for CMRA versus the HMA base layer. In particular, the substitution of natural aggregates with RAP lowered the emissions in water in terms of nitrogen and phosphorous compounds emitted during natural aggregates’ production and supply to the asphalt plant; furthermore, the asphalt materials that showed the best synergy between the minimization of environmental impacts and costs and maximization of the service life of the pavement solutions were the PMA combined with the base layer HMAJ, increasing the service life of a traditional HMA stratigraphy by 11 years.

From the point of view of the potential impacts of the present work in the road industry, decision-makers and road managing bodies could benefit from the use of the developed methodology and application; the results of the work represent a necessary innovation to comply with the future needs of both local legislative frameworks regarding the mandatory application of building information modeling to public bidding processes and the adoption of minimum environmental criteria to spread sustainability across the conception, design and operational stages of the life cycle of infrastructure.

Further efforts will be dedicated to the inclusion of an uncertainty analysis, which investigates the uncertainty of the input variables and its effect on the outcomes of the decision-making problem, and to the integration of social aspects through the application of a social LCA to complement the LCA and LCCA results and the extension of the problem of life-cycle-based road maintenance management to the network level.

## Figures and Tables

**Table 1:** Overview of the equations adopted to automate life cycle assessment calculations.

Life Cycle Phase	Equations
General equation of LCA	$EI_{x}=EI_{x}^{RMP}+EI_{x}^{A}+EI_{x}^{CON}+EI_{x}^{MN}+EI_{x}^{EOL}$
Raw materials production	$EI_{x}^{RMP}=\sum_{i=1}^{a}\sum_{j=1}^{b}(Q_{i,j}^{M}\cdot EI_{j,x}^{M}+Q_{i,j}^{M}\cdot D_{j}^{M}\cdot EI_{j,x}^{T})$
Asphalt mixture in-plant production	$EI_{x}^{A}=\sum_{i=1}^{a}f_{i}\cdot \left(Q_{i}^{A}\cdot EI_{x}^{AP}+Q_{i}^{A}\cdot D^{A}\cdot EI_{x}^{T}\right)$
Road pavement construction	$EI_{x}^{CON}=\sum_{i=1}^{a}\sum_{k=1}^{c}\left[\frac{Q_{i}^{A,ES}}{P^{M}}\cdot EI_{k,x}^{M}+Q_{i}^{A,ES}\cdot P_{i}^{W}\cdot EI_{x}^{W}+Q_{i}^{A,ES}\cdot \left(1-P_{i}^{W}\right)\cdot EI_{x}^{R}+\frac{Q_{i}^{A}}{P_{i,k}^{E}}\cdot EI_{k,x}^{C}\right]$
Road pavement maintenance	$EI_{x}^{MN}=\sum_{i=1}^{a}\left[\left(EI_{i,x}^{RMP}+EI_{i,x}^{A}+EI_{i,x}^{C}\right)\cdot m_{i}\right]$
End-of-life	$EI_{x}^{EOL}=\sum_{i=1}^{a}\left[Q_{i}^{A}\cdot D^{W}\cdot EI_{x}^{T}+Q_{i}^{A}\cdot P_{i}^{W}\cdot EI_{x}^{W}+Q_{i}^{A}\cdot \left(1-P_{i}^{W}\right)\cdot EI_{x}^{R}\right]$

*a* is a counter that refers to the specific asphalt layer of the pavement and is included in the range [1,3]; *b* is a counter of the raw materials included in each mixture. The range of variation of *b* varies according to the alternative mixtures and their relative components. In the present work, *b* is in the range [1,8]; *c* is a counter of the number of operating pieces of equipment for both milling, laying and compaction of HMAs and cold in-place recycling of CMRAs. In the present work, c is in the range [1,9]. $EI_{x}$ is the *x*-th environmental indicator (*EI*) of the whole life cycle of the system under analysis. $EI_{x}^{RMP}$, $EI_{x}^{A}$, $EI_{x}^{CON}$ and $EI_{x}^{MN}$ are, respectively, the *x*-th EIs of the production and supply of raw materials, in-plant asphalt mixture manufacturing and supply, pavement construction and pavement maintenance and end-of-life. $EI_{j,x}^{M}$ is the *x*-th *EI* related to the production of 1 tonne of the *j*-th raw material; $EI_{j,x}^{T}$ is the *x*-th *EI* of the transportation of 1 tonne of the *j*-th material for 1 km (fine aggregates are hauled by tanker trucks, while coarser aggregates and asphalt mixtures are hauled by dump trucks); $Q_{i,j}^{M}$ is the mass of the *j*-th raw material in the *i*-th asphalt layer (t); $D_{j}^{M}$ is the distance of the production facility of the *j*-th raw material from the asphalt plant (km); $Q_{i}^{A}$ is the mass of asphalt mixture produced to build the *i*-th pavement layer (t); $f_{i}$ is a dummy variable, equal to 1 when the *i*-th asphalt mixture is produced in the asphalt plant and 0 if the asphalt mixture is produced in place; $EI_{x}^{AP}$ is the *x*-th *EI* related to the in-plant manufacturing process of 1 tonne of asphalt mixture; $D^{A}$ is the distance of the asphalt production from the construction site (km); $Q_{i}^{A,ES}$ is the volume of the existing *i*-th asphalt layer to be milled (m^3^); $P^{M}$ is the productivity of the milling operations, where machineries work in series (m^3^/h); $EI_{k,x}^{M}$ is the *x*-th *EI* of the *k*-th milling equipment (milling machine and dump truck) for each operating hour; $P_{i}^{W}$ is the percentage by mass of the *i*-th existing milled asphalt layer that is disposed in landfill; $EI_{x}^{W}$ is the *x*-th *EI* for 1 tonne of milled asphalt pavement disposed in the landfill; $EI_{x}^{R}$ is the *x*-th *EI* for 1 tonne of milled asphalt pavement recycled as RAP; $P_{i,k}^{E}$ is the productivity of the *k*-th construction equipment used to build the *i*-th asphalt layer (grader, paver and roller for hot construction; grader, pulvimixer, steel and pneumatic rollers for cold recycled layers) (t, m^2^ or m^3^/h); $EI_{k,x}^{C}$ is the *x*-th *EI* of the *k*-th construction equipment for each operating hour; $m_{i}$ is the number or maintenance interventions in the analysis period; and $D^{W}$ is the distance from the construction site to the landfill (km).

**Table 2:** Overview of the equations adopted to automate life cycle cost analysis calculations.

Item	Equations
General equation of LCCA	$LCCA=C^{CON}+C^{MN}+C^{EOL}-S\frac{1}{{\left(1+r\right)}^{T}}$
Salvage Value	$S=\left(1-\frac{L_{S}}{L_{A}}\right)\cdot C^{MN,\;last}$
In-plant asphalt mixture production and supply, road pavement construction	$C^{CON}=\sum_{i=1}^{a}\left(\sum_{j=1}^{b}Q_{i,j}^{M}\cdot C_{j}^{M}+\sum_{k=1}^{c}\left(\frac{Q_{i}^{A}}{P_{i,k}^{E}}\cdot C_{k}^{E}+\sum_{l=1}^{d}\frac{Q_{i}^{A}}{P_{i,k,l}^{E}}\cdot n_{k,l}^{W}C_{l}^{W}\right)\right)$
Road pavement maintenance	$C^{MN}=\sum_{i=1}^{a}\left[\left(\sum_{j=1}^{b}Q_{i,j}^{M}\cdot C_{j}^{M}+\sum_{k=1}^{c}\left(\frac{Q_{i}^{A}}{P_{i,k}^{E}}\cdot C_{k}^{E}+\sum_{l=1}^{d}\frac{Q_{i}^{A}}{P_{i,k,l}^{E}}\cdot n_{k,l}^{W}C_{l}^{W}\right)\right)\cdot m_{i}\cdot \frac{1}{{\left(1+r\right)}^{n_{i}}}\right]$
End-of-life	$C^{EOL}=\sum_{i=1}^{a}\left[\left(Q_{i}^{A}\cdot D^{W}\cdot C^{T}\right)+\left(Q_{i}^{A}\cdot P_{i}^{W}\cdot C^{W}\right)+\left(Q_{i}^{A}\cdot \left(1-P_{i}^{W}\right)\cdot C^{R}\right)\right]\cdot \frac{1}{{\left(1+r\right)}^{n}}$

$C^{CON}$ is the net present value of the construction cost of the pavement (EUR); $C^{MN}$ is the net present value of the maintenance cost of the pavement (EUR); $C^{EOL}$ is the net present value of the end of life cost of the pavement (EUR); *T* is the analysis period (y); *r* is the discount rate; $L_{S}$ is the expected service life of the last maintenance intervention (y); $L_{A}$ is analysis life of the last maintenance intervention, i.e., difference between the year of construction of the maintenance intervention and the year of termination of the analysis period (y); $C^{MN,last}$ is the cost of the last maintenance intervention (EUR); S is the salvage value of the pavement at the end of the analysis period (EUR); $C_{j}^{M}$ is the unit supply cost of the *j*-th raw material (EUR/t); $C_{k}^{E}$ is the hourly cost of the *k*-th construction equipment (EUR/h); $P_{i,k,l}^{E}$ is the is the productivity of the *k*-th construction equipment used to build the *i*-th asphalt layer referred to the *l*-th category of workers (skilled, specialized, etc.) (t, m^2^ or m^3^/h/worker); $n_{k,l}^{W}$ is the number of workers of the *l*-th category required to handle the *k*-th construction equipment; $C_{l}^{W}$ is the hourly cost of the *l*-th category of workers (EUR/h); $m_{i}$ is the number of maintenance interventions involving the reconstruction of the *i*-th asphalt layer during the analysis period; and $n_{i}$ is the number of years after which a maintenance intervention should be carried out (y). The meaning of all the missing parameters is reported in Table 1.

## Data Availability

Data are contained within the article or Supplementary Materials or are available on request from the corresponding author.

## Supplementary Materials

The following supporting information can be downloaded at: https://www.mdpi.com/article/10.3390/ma16031047/s1, Table S1: Mix composition of the hot asphalt mixtures for the binder layer; Table S2: Mix composition of the asphalt mixtures for the base layer; Table S3: Indirect tensile stiffness modulus results at 5, 10, 20 and 30 °C for the asphalt mixtures under analysis; Table S4: Traffic and load data for asphalt pavement design; Table S5: Overview of the maintenance alternatives considered for the application of the methodology; Table S6: Overview of the data sources for life cycle assessment; Table S7: Results of LCCA as obtained from the IBIM analysis tool; Table S8: Decision matrix of the case study as obtained from the IBIM tool.
