# Supplementary material for: Leveraging Infrastructure BIM for Life-Cycle-Based Sustainable Road Pavement Management
Source: Materials (Basel). 2023 Jan 24;16(3):1047. doi: 10.3390/ma16031047 (PMC9920642; doi:10.3390/ma16031047)
Supplement: Supplementary file 1 [file materials-16-01047-s001.zip › materials-2141726-supplementary.pdf]

Summary of content:

Table S1. Mix composition of the hot asphalt mixtures for the binder layer.

Table S2. Mix composition of the asphalt mixtures for the base layer.

Table S3. Indirect tensile stiffness modulus results at 5, 10, 20, and 30 °C for the asphalt mixtures under analysis.

Table S4. Traffic and load data for asphalt pavement design.

Table S5. Overview of the maintenance alternatives considered for the application of the methodology

Table S6. Overview of the data sources for Life Cycle Assessment

Table S7. Results of LCCA as obtained from the IBIM analysis tool

Table S8. Decision matrix of the case study as obtained from the IBIM tool

Table S1. Mix composition of the hot asphalt mixtures for the binder layer.

Mixture ID	Properties	HMA	HMAC	HMMA	PMA
Mix composition	Limestone 12/18 mm	25%	23%	25%	25%
	Limestone 6/12 mm	33%	29%	33%	33%
	Limestone 3/6 mm	–	13%		
	Limestone sand	38%	31%	38%	38%
	Limestone filler	4%	–	4%	4%
	CDW	–	4%	–	–
	Bitumen wa.% *	5.00%	5.75%	5.00%	5.00%
	Polymer pellets wb.% **	–	–	–	5.00%
Volumetric properties	% air voids	4.00%	4.20%	4.00%	4.20%
	Specific gravity, g·cm^{-3}	2.52	2.48	2.50	2.50

Table S2. Mix composition of the asphalt mixtures for the base layer.

Mixture ID	Properties	HMA	HMAJ	CMRA
Mix composition	Limestone 18/31.5 mm	9%	9%	16%
	Limestone 12/18 mm	32%	32%	7%
	Limestone 6/12 mm	31%	31%	–
	Limestone sand	21%	21%	–
	Limestone filler	7%	–	7%
	JGW	–	7%	-
	RAP	–	–	70%
	Bitumen wa.%	4.50%	4.85%	–
	Bituminous emulsion wa.%	–	–	3.75%
	Cement wa.%	–	–	1.50%
Volumetric properties	Air voids	4.00%	3.90%	9.00%
	Specific gravity, g·cm^{-3}	2.35	2.37	2.49

Table S3. Indirect tensile stiffness modulus results at 5, 10, 20, and 30 °C for the asphalt mixtures under analysis.

Asphalt Layer	Mixture Identification	ITSM (MPa) – EN 12697-26 Annex C 5 °C	10 °C	20 °C	30 °C	ν
Wearing course	HMA	10013	6931	3077	1355	0.35
Binder layer	HMA	12780	8279	5299	1818	0.35
	HMAC	11921	8120	5301	1977	0.35
	HMMA	25136	15391	8613	3402	0.35
	PMA	31143	16498	9582	2897	0.35
Base layer	HMA	15500	10220	5952	2960	0.35
	HMAJ	17649	11725	6826	3204	0.35
	CMRA	9101	6380	3267	2203	0.35

Table S4. Traffic and load data for asphalt pavement design.

Parameter	Units	Value
Road category	-	C
AADT - Annual Average Daily Traffic	1/d	5000
Rate of heavy vehicles	%	5
R – Traffic growth rate	%	3
Traffic spectrum	-	Road category C traffic spectrum
ESALS in 20 years	-	1′633′384

Table S5. Overview of the maintenance alternatives considered for the application of the methodology.

Type of maintenance intervention	Description	Expected frequency of maintenance interventions	Materials' alternatives in the case study
Local repairs	Potholes repair (patching)	1-2 years	Asphalt mixture depending on the pothole depth
	Crack sealing	1-2 years	Asphalt sealants
Surface rehabilitation	Milling and reconstruction of the wearing course	3-5 years	HMA – wearing course
Deep rehabilitation	Milling and reconstruction of the wearing course and binder layer	5-15 years	HMA – wearing course<br>HMA – binder layer<br>HMAC – binder layer<br>PMA – binder layer<br>HMMA – binder layer
Reconstruction	Milling and reconstruction of the asphalt layers (base, binder and wearing course)	10-30 years	HMA – wearing course<br>HMA – binder layer<br>HMAC – binder layer<br>PMA – binder layer<br>HMMA – binder layer<br>HMA – base layer<br>CMRA – base layer<br>HMAJ – base layer

Table S6. Overview of the data sources for Life Cycle Assessment.

Phase of the life cycle	Unit process	Primary data	Year of survey	Secondary data
Aggregates production	coarse limestone aggregates	Amount in the asphalt mixtures	2019/2020	Ecoinvent 3 database: Gravel, crushed {RoW}∣ production ∣ Cut-off, U
	coarse basalt aggregates			Ecoinvent 3 database: Basalt {RER}∣ quarry operation ∣ Cut-off, U
	limestone sand			Ecoinvent 3 database: Sand {RoW}∣ gravel and quarry operation ∣ Cut-off, U
	limestone filler			Ecoinvent 3 database: Lime, packed {Europe without Switzerland}∣ lime production, milled, packed ∣ Cut-off, U
Bituminous binders production	neat bitumen<br>SBS-modified bitumen<br>bitumen emulsion	Amount in the asphalt mixtures	2019/2020	The Eurobitume Life-Cycle Inventory for Bitumen, Version 3.1, European Bitumen Association, Brussels, Belgium (2020)
Cement production	cement production	Amount in the cold asphalt mixtures	2019/2020	Ecoinvent 3 database: Cement, Portland {Europe without Switzerland}∣ market for ∣ Cut-off, U
Recycled polymer pellets production	Plastic waste recycling and shredding	Amount in the asphalt mixture	2019/2020	Ecoinvent 3 database: Polyethylene terephthalate, granulate, bottle grade, recycled {RoW}∣ polyethylene terephthalate production, granulate, bottle grade, recycled ∣ Cut-off, U
	Pelletisation			Santos et al. (2021)
CDW management	Waste concrete treatment facility	Amount in the asphalt mixture	2019/2020	Ecoinvent 3: Waste concrete gravel {RoW}∣ treatment of waste concrete gravel, recycling ∣ Cut-off, U
JGW management	JGW collection	Amount of JGW produced in the construction site (210 t) Productivity of the shovel (24.8 m^3/h)	2019	Ecoinvent 3 database: Machine operation, diesel, >= 74.57 kW, steady-state {GLO}∣ market for ∣ Cut-off, U (Crushing of JGW)
	JGW crushing	Amount in the asphalt mixtures<br>Productivity of the jaw mill (150 t/h)	2019/2020	Ecoinvent 3 database: Machine operation, diesel, >= 74.57 kW, steady-state {GLO}∣ market for ∣ Cut-off, U (Crushing of JGW)
RAP management	RAP collection	Amount of RAP produced in the construction site (210 t)	2019	Ecoinvent 3: Waste asphalt {RoW}∣ treatment of, sanitary landfill ∣ Cut-off, U
Hot mix asphalt production	Asphalt plant infrastructure			Ecoinvent 3 database: Industrial machine, heavy, unspecified {GLO}∣ market for ∣ Cut-off, U
	Wheel loader for aggregates moving	Amount of aggregates in the hot mix asphalt<br>Productivity of the wheel loader (70 t/h)	2019/2020	Ecoinvent 3 database: Machine operation, diesel, >= 74.57 kW, steady-state {GLO}∣ market for ∣ Cut-off, U
	Conveyor belt	Amount of aggregates in mixture	2019/2020	Ecoinvent 3 database: Conveyor belt {GLO}∣ market for ∣ Cut-off, U
	Aggregates drying	Amount of asphalt mixture<br>Unit natural gas consumption (8.79 m3/t of asphalt mix)	2019/2020	Ecoinvent 3 database: Drying, natural gas {GLO}∣ market for ∣ Cut-off, U
	Asphalt mixing	Amount of asphalt mixture.<br>Unit electricity consumption = 4.37 kWh/t of asphalt mix	2019/2020	Ecoinvent 3 database: Electricity, medium voltage {IT}∣ market for ∣ Cut-off, U<br>United States Environmental Protection Agency: emissions of Benzo(a)pyrene = 4.66E-10 kg/t<br>United States Environmental Protection Agency: emissions of NMVOC = 0.00019 kg/
Pavement construction with hot mix asphalt	Laying of hot mix asphalt	Amount of asphalt mixture productivity of machinery = 351 t/h (wearing course), 205 t/h (binder layer), 117 t/h (base layer)	2021	Ecoinvent 3 database: Machine operation, diesel, >= 74.57 kW, steady-state {GLO}∣ market for ∣ Cut-off, U (Paver)
	Compaction of hot mix asphalt			Ecoinvent 3 database: Machine operation, diesel, >= 18.64 kW and < 74.57 kW, steady-state {GLO}∣ market for ∣ Cut-off, U (Roller)
Base layer construction with cold in-place recycling technique	Surface levelling and RAP placing over the pavement surface	Amount of cold asphalt mixture Grader productivity = 545 m2/h	2019/2020	Ecoinvent 3 database: Machine operation, diesel, >= 74.57 kW, steady-state {GLO}∣ market for ∣ Cut-off, U (Grader)
	Mixing of components and laying of cold mix asphalt	Pulvimixer productivity = 750m2/h	2021	Ecoinvent 3 database: Machine operation, diesel, >= 74.57 kW, steady-state {GLO}∣ market for ∣ Cut-off, U (Pulvimixer)
	Compaction of cold mix asphalt	3 rollers, productivity = 141 t/h	2021	Ecoinvent 3 database: Machine operation, diesel, >= 74.57 kW, steady-state {GLO}∣ market for ∣ Cut-off, U (Roller 1)<br>Ecoinvent 3 database: Machine operation, diesel, >= 18.64 kW and < 74.57 kW, steady-state {GLO}∣ market for ∣ Cut-off, U (Roller 2)<br>Ecoinvent 3 database: Machine operation, diesel, >= 18.64 kW and < 74.57 kW, steady-state {GLO}∣ market for ∣ Cut-off, U (Roller 3)
End of life	Demolition of the pavement	Productivity of the milling machine = 150 t/h	2020	Ecoinvent 3 database: Machine operation, diesel, >= 74.57 kW, steady-state {GLO}∣ market for ∣ Cut-off, U (Milling machine)
	Disposal in landfill	Amount of landfilled asphalt waste	2019	Ecoinvent 3 database: Waste asphalt {RoW}∣ treatment of, sanitary landfill ∣ Cut-off, U
Transportation	Transport by ship	Covered distance. Amount of materials (raw materials, asphalt mixtures, asphalt waste) to be supplied	2019/ 2020	Ecoinvent 3 database: Transport, freight, sea, tanker for liquid goods other than petroleum and liquefied natural gas {GLO}∣ market for transport, freight, sea, tanker for liquid goods other than petroleum and liquefied natural gas ∣ Cut-off, U
	Transport by road		2019/ 2020	Ecoinvent 3 database: Transport, freight, lorry 16-32 metric ton, euro4 {RER}∣ market for transport, freight, lorry 16-32 metric ton, EURO4 ∣ Cut-off, U

Table S7. Results of LCCA as obtained from the IBIM analysis tool.

n°	Type of approach	Pavement solution	LCC without considering the salvage value	Salvage value	LCC indicator
1	Reconstruction	HMA-HMA-HMA	1,394,143.7 €	36,570.5 €	1,357,573.2 €
2	Reconstruction	HMA-PMA-HMA	1,279,211.7 €	98,897.1 €	1,180,314.6 €
3	Reconstruction	HMA-HMMA-HMA	1,300,240.7 €	79,719.6 €	1,220,521.1 €
4	Reconstruction	HMA-HMAC-HMA	1,307,867.5 €	57,985.6 €	1,249,881.8 €
5	Reconstruction	HMA-HMA-CMRA	1,025,674.8 €	6,982.3 €	1,018,692.5 €
6	Reconstruction	HMA-PMA-CMRA	946,954.2 €	87,554.5 €	859,399.6 €
7	Reconstruction	HMA-HMMA-CMRA	979,180.1 €	60,034.9 €	919,145.1 €
8	Reconstruction	HMA-HMAC-CMRA	962,464.6 €	59,010.1 €	903,454.5 €
9	Reconstruction	HMA-HMA-HMAJ	902,280.9 €	108,807.7 €	793,473.1 €
10	Reconstruction	HMA-PMA-HMAJ	751,723.5 €	33,210.3 €	718,513.2 €
11	Reconstruction	HMA-HMMA-HMAJ	770,452.4 €	9,236.1 €	761,216.3 €
12	Reconstruction	HMA-HMAC-HMAJ	882,517.6 €	118,020.2 €	764,497.4 €
13	Rehabilitation	HMA-HMA	1,357,055.4 €	36,758.5 €	1,320,296.9 €
14	Rehabilitation	HMA-PMA	1,011,163.0 €	6,597.7 €	1,004,565.4 €
15	Rehabilitation	HMA-HMMA	981,135.2 €	28,589.9 €	952,545.2 €
16	Rehabilitation	HMA-HMAC	1,175,187.7 €	19,060.0 €	1,156,127.8 €

Table S8. Decision matrix of the case study as obtained from the IBIM tool.

Pavement solution	Units	HMA-HMA-HMA	HMA-PMA-HMA	HMA-HMMA-HMA	HMA-HMAC-HMA	HMA-HMA-CMRA	HMA-PMA-CMRA	HMA-HMMA-CMRA	HMA-HMAC-CMRA	HMA-HMA-HMAJ	HMA-PMA-HMAJ	HMA-HMMA-HMAJ	HMA-HMAC-HMAJ	HMA-HMA	HMA-PMA	HMA-HMMA	HMA-HMAC
R	mm	21.1966	1.3608	1.9282	16.1826	12.305	0.6884	0.5841	0.1748	1.521	0.7635	18.12	22	15.645	16.12	5.01	13.01
FD	-	0.81	0.56	0.51	0.46	0.82	0.1495	0.2916	0.27	0.4496	0.1634	0.8301	1	0.7288	0.11	0.07	0.16
PCI	-	60	-	-	-	-	-	-	-	-	-	-	-	59	-	-	-
GWP	kg CO2 eq	1.29E+06	1.08E+06	1.14E+06	1.20E+06	1.04E+06	8.36E+05	9.25E+05	8.96E+05	9.44E+05	7.87E+05	8.81E+05	8.99E+05	1720274.6	997273.89	946543.18	1429456.904
ODP	kg CFC11 eq	5.33E-01	6.28E-01	6.44E-01	5.70E-01	5.87E-01	5.12E-01	5.46E-01	5.97E-01	5.60E-01	6.77E-01	7.10E-01	5.39E-01	0.7101022	0.4105362	0.3857556	0.590057207
IR	kBq Co-60 eq	5.68E+04	6.87E+04	7.01E+04	6.07E+04	6.57E+04	5.39E+04	6.01E+04	6.21E+04	6.00E+04	7.25E+04	7.60E+04	5.76E+04	75675.815	44665.822	41776.632	62882.58657
OFH	kg NOx eq	6.78E+03	8.03E+03	8.33E+03	7.26E+03	7.63E+03	6.74E+03	7.16E+03	8.01E+03	7.37E+03	8.90E+03	9.43E+03	7.08E+03	9040.5165	5246.2448	4969.3242	7512.189479
PM	kg PM2.5 eq	1.00E+04	1.21E+04	1.26E+04	1.07E+04	8.18E+03	7.37E+03	7.73E+03	8.59E+03	1.11E+04	1.34E+04	1.41E+04	1.06E+04	13353.14	7891.0948	7459.1971	11095.7511
OFT	kg NOx eq	7.38E+03	8.75E+03	9.07E+03	7.89E+03	8.13E+03	7.18E+03	7.62E+03	8.53E+03	8.03E+03	9.70E+03	1.03E+04	7.71E+03	9831.1039	5712.7273	5409.4291	8169.125589
A	kg SO2 eq	4.79E+03	5.70E+03	5.97E+03	5.12E+03	5.77E+03	5.03E+03	5.51E+03	6.06E+03	5.25E+03	6.35E+03	6.81E+03	5.04E+03	6384.6896	3718.5306	3547.6046	5305.338253
FE	kg P eq	1.76E+02	2.14E+02	2.18E+02	1.89E+02	2.04E+02	1.62E+02	1.91E+02	2.14E+02	1.87E+02	2.28E+02	2.37E+02	1.80E+02	235.26383	139.32198	129.83593	195.4917581
ME	kg N eq	1.59E+03	1.94E+03	2.00E+03	1.70E+03	1.76E+03	1.56E+03	1.63E+03	1.85E+03	1.76E+03	2.13E+03	2.24E+03	1.69E+03	2119.8758	1257.2609	1186.8151	1761.50429
TECO	kg 1,4-DCB	1.02E+07	1.15E+07	1.18E+07	1.09E+07	1.02E+07	9.17E+06	9.53E+06	1.07E+07	1.04E+07	1.25E+07	1.31E+07	9.96E+06	13639255	7629157.9	7176554.7	11333497.43
FECO	kg 1,4-DCB	4.78E+04	5.78E+04	5.88E+04	5.11E+04	5.23E+04	4.65E+04	4.86E+04	5.49E+04	5.14E+04	6.27E+04	6.52E+04	4.94E+04	63718.874	37597.928	35074.833	52947.00289
MECO	kg 1,4-DCB	6.87E+04	8.27E+04	8.42E+04	7.35E+04	7.47E+04	6.65E+04	6.94E+04	7.84E+04	7.36E+04	8.97E+04	9.33E+04	7.07E+04	91619.692	53834.425	50264.811	76131.10186
CT	kg 1,4-DCB	3.57E+04	4.29E+04	4.37E+04	3.82E+04	3.87E+04	3.36E+04	3.61E+04	4.06E+04	3.80E+04	4.61E+04	4.82E+04	3.65E+04	47597.65	27949.231	26110.942	39551.12114
NCT	kg 1,4-DCB	8.34E+05	9.95E+05	1.01E+06	8.92E+05	9.02E+05	7.96E+05	8.41E+05	9.48E+05	8.75E+05	1.07E+06	1.11E+06	8.41E+05	1111894.5	649004.63	603694.77	923925.3599
LU	m2a crop eq	1.15E+05	1.38E+05	1.42E+05	1.23E+05	1.34E+05	1.19E+05	1.24E+05	1.41E+05	1.22E+05	1.47E+05	1.55E+05	1.17E+05	153100.19	89815.339	84626.625	127218.1357
MR	kg Cu eq	4.50E+03	5.33E+03	5.44E+03	4.81E+03	5.50E+03	4.86E+03	5.08E+03	5.77E+03	4.75E+03	5.75E+03	6.02E+03	4.56E+03	5994.1223	3482.6536	3257.7976	4980.79752
FR	kg oil eq	1.19E+06	1.42E+06	1.48E+06	1.27E+06	1.56E+06	1.36E+06	1.45E+06	1.64E+06	1.39E+06	1.68E+06	1.78E+06	1.33E+06	1580071	925835.96	877974.78	1312955.113
W	m3	2.06E+04	2.52E+04	2.67E+04	2.20E+04	2.31E+04	2.04E+04	2.25E+04	2.43E+04	2.22E+04	2.68E+04	2.91E+04	2.13E+04	27468.742	16324.641	15686.942	22825.06678
LCC indicator	€	1.36E+06	1.18E+06	1.22E+06	1.25E+06	1.02E+06	8.59E+05	9.19E+05	9.03E+05	7.93E+05	7.19E+05	7.61E+05	7.64E+05	1320296.9	1004565.4	952545.23	1156127.76
